# Field Map Reconstruction in Magnetic Resonance Imaging Using Bayesian Estimation

**DOI:** 10.3390/s100100266

**Published:** 2009-12-30

**Authors:** Fabio Baselice, Giampaolo Ferraioli, Aymen Shabou

**Affiliations:** 1 Dipartimento per le Tecnologie, Università degli Studi di Napoli Parthenope, Naples, Italy; E-Mail: giampaolo.ferraioli@uniparthenope.it; 2 Institut TELECOM, TELECOM ParisTech, CNRS LTCI, Paris, France; E-Mail: aymen.shabou@telecom-paristech.fr

**Keywords:** Magnetic Resonance Imaging, field map estimation, phase unwrapping, bayesian estimation, graph-cuts, Markov Random Field

## Abstract

Field inhomogeneities in Magnetic Resonance Imaging (MRI) can cause blur or image distortion as they produce off-resonance frequency at each voxel. These effects can be corrected if an accurate field map is available. Field maps can be estimated starting from the phase of multiple complex MRI data sets. In this paper we present a technique based on statistical estimation in order to reconstruct a field map exploiting two or more scans. The proposed approach implements a Bayesian estimator in conjunction with the Graph Cuts optimization method. The effectiveness of the method has been proven on simulated and real data.

## 1. Introduction

Magnetic Resonance Imaging (MRI) is a coherent imaging technique consisting of detecting signals induced by nuclei of the object being imaged in complex domain. To allow nuclei to produce signals, the object has to be placed in a uniform magnetic field and sequentially excited with suitable RF impulses.

Some imaging techniques show high sensitivity to the non uniformities of the applied magnetic field, particularly when exploiting long readout times, for example echo-planar imaging and spiral scans. The most primary effects of field inhomogeneities in MR images are blur and distortion. Such errors cannot be removed unless an accurate field map is available and used to compensate the complex data [1].

Field map can be estimated from different scans (at least two) acquired at different echo times. The phase difference between the acquired images is due to the different precession frequencies, which are related to the field map via a linear relation.

Besides the trivial estimation consisting of dividing the phase difference by the delay time between acquisitions *Δ_TE_*, some more sophisticated procedures can be proposed. In literature two approaches have been presented exploiting two or more MR complex images: statistical and non |statistical approaches.

Non statistical approaches are mainly based on retrieving the field map using linear regression techniques [2] or on standard phase unwrapping techniques consisting of adding multiples of 2*π* to phase data [3]. In both techniques Gaussian filtering is suggested in order to obtain a more accurate final reconstructed image. Note that these approaches have a main limit: since they are not statistically based, they do not exploit noise information for the estimation.

Statistical approaches are based on the exploitation of the noise statistics in order to obtain a more efficient estimation from the information theory point of view. A Penalized Maximum Likelihood Estimator exploiting two or more images (*i.e.*, multi-acquisition) has been presented in [4], showing the potentialities of the statistical approach. In [4], by introducing a quadratic form for the regularization term, the authors assume a smooth and homogeneous field map. Moreover, they use small echo time differences in order to prevent phase wrapping.

In this paper we propose a novel multi-acquisition Maximum *A Posteriori* (MAP) estimator which overcomes some limitations respect to other presented techniques, as it does not need any initialization while removing tight limitations on required *Δ_TE_*. This approach has been developed consequently to an accurate study on noise model in complex MRI data and in order to take into account the piecewise smooth nature of the field maps (smooth areas and strong local changes of the filed strength). The proposed algorithm works jointly on the multi-acquisition available phase images, allowing to automatically perform PU operation and correctly reconstruct smooth areas and field discontinuities (e.g., field discontinuities at air/tissue or fat/water boundaries).

In Section 2 the field map estimation problem is briefly addressed. In Section 3 the proposed method will be presented. The optimization algorithm will be explained in Section 4 and the results will be shown and discussed in Section 5. Finally, we draw some conclusions about the presented technique.

## 2. Problem Statement

In MRI the signal comes from nuclei with spin which rotate at a certain frequency, called the Larmor angular frequency. The precession depends on the kind of nuclei and the energy of the state in which the nuclei are in a magnetic field *B*_0_ [1]. Let us consider a complex MR image *x*_1_ taken at an echo time *T*_*E*,1_; the analytical expression of the complex MR image *x*_1_ is given by:

(1) $$x_{1}=m_{1}\;e^{i\phi_{1}}$$

where *m*_1_ represents the amplitude of the signal and *ϕ*_1_ the phase at the time *T*_*E*,1_; we can represent the phase *ϕ*_1_ as the sum of an initial phase *ϕ_0_* and a term related to Larmor angular frequency *ω*:

(2) $$\phi_{1}=\phi_{0}+\omega T_{E,1}$$

If we consider a second complex image taken at an echo time *T*_*E*,2_, the complex image will be:

(3) $$x_{2}=m_{2}\;e^{i\phi_{2}}$$

where *m*_2_ represents the amplitude of the signal and *ϕ*_2_:

(4) $$\phi_{2}=\phi_{0}+\omega T_{E,2}$$

Note that Equations (1) and (3) are an approximate model as they ignore the presence of off-resonance artifacts. This assumption requires that short echo times are used for the acquisition of the images and will be considered a valid approximation for the rest of the paper.

Using *x*_1_ and *x*_2_ we can estimate the Larmor angular frequency *ω* of each voxel (pixel of the MR image) computing the phase difference between *ϕ*_1_ and *ϕ*_2_ and dividing by *Δ_TE_* *=* (*T*_*E*,2_ – *T*_*E*,1_). Once obtained *ω*, the field map can be recovered exploiting the relation between Larmor angular frequency and the magnetic field *B*:

(5) ω=γB

where *γ* is a known parameter representing the gyromagnetic ratio which depends on the atomic nucleus.

This seemingly simple approach would generate properly reconstructed field maps if there are no 2*π* wraps in the (*ϕ*_2_ – *ϕ*_1_) phase data, otherwise phase unwrapping operation will be needed before computing *ω*. Note also that this trivial approach does not consider the complex noise added to signals *x*_1_ and *x*_2_.

## 3. Maximum *A Posteriori* Field Map Estimation

Let us consider *N* complex images ***x*** = [ *x*_1_, *x*_2_…*x_N_*]^T^ obtained at echo times *T_E_* = [*T*_*E*,1_, *T*_*E*,2_,…*T_E,N_*]^T^. By generalizing Equation (2), when different acquisitions are available, neglecting the initial phase *ϕ*_0_, the relation between the *n*-th phase and the Larmor angular frequency is given by:

(6) $$\phi_{n}=\omega T_{E,n}$$

Our idea is to perform the field map estimation exploiting jointly the phases of images *x*. In the Bayesian estimation framework, we propose a Maximum *A Posteriori* (MAP) solution for the estimation of the unknown parameter *ω*. We recall that MAP criterion consists of maximization of the *a posteriori* probability density function (pdf) which is, according to Bayes Law, proportional to the product of the likelihood function and the *a priori* pdf.

In order to obtain the likelihood function, we investigate the pdf of the involved noise. In the *k*-space domain, the signal coming from voxels is mixed with Additive White Gaussian Noise (AWGN) in both real and imaginary parts [5,6]. As the Fourier Transformation is a linear operator, in the complex image domain noise samples are still additive, gaussian and uncorrelated. Considering amplitude and phase of the complex signal, the noise pdf leads to a Rice distribution in terms of signal amplitude, and to the following distribution for the signal phase *ϕ* [5,6]:

(7) $$f\;(\phi )=\frac{1}{2\pi }\;e^{-\frac{A^{2}}{2\sigma^{2}}}\;[1+\frac{A}{\sigma }\text{cos}(\phi )\cdot e^{-\frac{A^{2}}{2\sigma^{2}}{\text{cos}}^{2}\;(\phi )}\;\int_{-\infty }^{\frac{A}{\sigma }\text{cos}(\phi )}\;e^{-\frac{x^{2}}{2}}\mathrm{dx}]$$

where *A* is the signal amplitude and *σ* is the noise standard deviation.

Expression (7) can be approximated well with the following probability density function [7]:

(8) $$f\;(\phi )=\frac{1-{\left|\gamma \right|}^{2}}{2\pi }\;\frac{1}{1-{\left|\gamma \right|}^{2}\;{\text{cos}}^{2}\;(\phi )}\;\left(1+\frac{\left|\gamma \right|\text{cos}(\phi )\;\text{arc cos}(-\left|\gamma \right|\;\text{cos}(\phi ))}{\sqrt{1-{\left|\gamma \right|}^{2}\;{\text{cos}}^{2}\;(\phi )}}\right)$$

where *γ* is the coherence of the signal which can be seen as a normalized Signal to Noise Ratio (SNR = *A*^2^/*σ*^2^). The relation between them is empirically found fixing an SNR and looking for the *γ* value minimizing the mean square error between the two pdfs.

**Figure 1.**
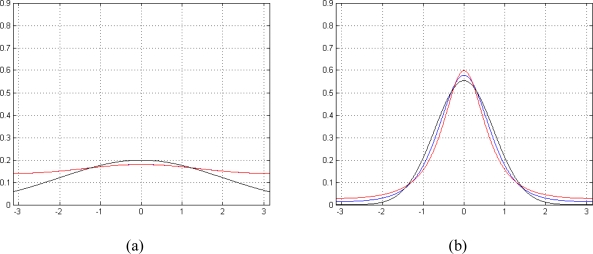
Probability density function of MRI phase signal noise, real pdf (blue), approximated pdf (red), Gaussian pdf (black): (a) low *γ* (0.08) and SNR (-20 dB) case (approximated pdf completely overlaps real one) and (b) high *γ*(0.75) and SNR (4 dB) case.

Figure 1 shows a comparison between (7) and (8). When *γ* for (8) or SNR for (7) is low, both pdfs tend to a uniform distribution in [-*π*,*π*] (Figure 1a), while, in case of higher *γ* or SNR, Functions (7) and (8) approach a Gaussian distribution (Figure 1b). Note that approximating Equation (7) with (8) leads to a more appropriate model compared to classical Gaussian approximation for MRI phase signal noise; as a matter of fact, the latter is a good approximation only in case of higher SNRs. As Equation (8) avoids the integration, we use for the rest of the paper Equation (8) instead of (7) in order to simplify the model and to have a lower computational cost.

The likelihood function can be obtained starting from the pdf (8) (or from Equation (7) at a higher algorithm computational cost) of an MRI phase image. Let us consider the *p*-th pixel of the image. Given *ϕ̆_p_* the measured noisy phase value and *ϕ_p_* the true phase, the pdf is given by [8,9]:

(9) $$l\left({\overset{⌣}{\phi }}_{p},\phi_{p}\right)=\frac{1-{{|\gamma }_{p}|}^{2}}{2\pi }\frac{1}{1-{|\gamma_{p}|}^{2}{\text{cos}}^{2}\left({\overset{⌣}{\phi }}_{p}-\phi_{p}\right)}\left(1+\frac{\left|\gamma_{p}\right|\text{cos}\left({\overset{⌣}{\phi }}_{p}-\phi_{p}\right)\;\text{arc cos}\left(-\left|\gamma_{p}\right|\text{cos}\left({\overset{⌣}{\phi }}_{p}-\phi_{p}\right)\right)}{\sqrt{1-{\left|\gamma_{p}\right|}^{2}{\text{cos}}^{2}\left({\overset{⌣}{\phi }}_{p}-\phi_{p}\right)}}\right)$$

Considering *N* acquisitions with independent noise samples obtained at different *T_E,n_*, the multi-acquisition likelihood function for the *p*-th pixel is the product of marginal ones (9), once Equation (6) is substituted in (9) [8]:

(10) $$\begin{array}{l} L\left({\overset{⌣}{\phi }}_{p}|\omega_{p}\right)=\prod_{n=1}^{N}\frac{1-{\left|\gamma_{p,n}\right|}^{2}}{2\pi }\frac{1}{1-{\left|\gamma_{p,n}\right|}^{2}{\text{cos}}^{2}\left({\overset{⌣}{\phi }}_{p,n}-\omega_{p}T_{E,n}\right)}\times \\ \;\;\;\;\;\;\;\left(1+\frac{\left|\gamma_{p,n}\right|\text{cos}\left({\overset{⌣}{\phi }}_{p,n}-\omega_{p}T_{E,n}\right)\;\text{arc cos}\left(-\left|\gamma_{p,n}\right|\text{cos}\left({\overset{⌣}{\phi }}_{p,n}-\omega_{p}T_{E,n}\right)\right)}{\sqrt{1-{\left|\gamma_{p,n}\right|}^{2}{\text{cos}}^{2}\left({\overset{⌣}{\phi }}_{p,n}-\omega_{p}T_{E,n}\right)}}\right) \end{array}$$

where *p*,*n* indexes stand for *p*-th pixel and *n*-th acquisition and ***ϕ̆****_p_* *=* [*ϕ̆_p,_*_1_, *ϕ̆_p,_*_2_, ⋯ *ϕ̆_p,N_*]*^T^* is the vector collecting the measured phases for the *N* acquisitions relative to pixel *p*.

Let us now consider the *a priori* pdf of the unknown parameter ω. We model it as a Markov Random Field (MRF). Thanks to Hammersley-Clifford theorem, any MRF can be expressed in terms of Gibbs distribution [10]. So the *a priori* pdf can be modelled by:

(11) $$f_{\Omega }\;(\omega )=\frac{1}{Z(\beta )}\;\text{exp}(-U(\omega ,\beta ))$$

where *U* is the priori energy function, *β* is the so called hyperparameter, which is used to tune the model, and ***ω*** = [*ω*_1_ *ω*_2_ *ω*_3…_*ω_P_*]^T^ is the collection of the Larmor angular frequencies related to the *P* pixels of the image. We choose the Total Variation (TV) model for the *a priori* energy function [11]:

(12) $$U\left(\omega ,\beta \right)=\sum_{p=1}^{P}\sum_{q\in {𝒩}_{P}}\beta \left|\omega_{p}-\omega_{q}\right|$$

where *𝒩_p_* is the neighborhood of the pixel *p* (the 4 nearest pixels).We choose the TV model since it looks for an approximation of the original noisy image which has minimal total variation but without particular bias to discontinuity or smooth solution [12].

Given the likelihood Function (10) and given the *a priori* pdf (12), the MAP solution is given by the following maximization:

(13) $${\overset{⌢}{\omega }}_{\mathrm{MAP}}=\text{arg}\;\underset{\omega }{\text{max}}\left[\left(\prod_{p=1}^{P}L\left({\overset{⌣}{\phi }}_{p,}\omega_{p}\right)\right)\;f_{\Omega }(\omega )\right]$$

Once this maximization has been performed, the field map for the whole image can be computed by simply inverting Equation (5). The details about the used maximization procedure will be discussed in the following section.

If the likelihood Function (10) shows more than one maximum, in order to obtain the uniqueness of the solution of the multi-acquisition Phase Unwrapping problem, the single acquisition likelihood functions need to have different periods, which is achieved considering a not rational value for the ratio between *T_E_* values [8].

## 4. Maximization Procedure

In order to obtain the field map estimation, Equation (13) needs to be solved. The maximization of the *a posteriori* distribution can turn out to be a difficult task. Due to the periodicity of the Likelihood function, Equation (13) can show more than one relative maximum that makes mandatory the use of a global optimization algorithm (*i.e.*, an optimization algorithm that is able to provide the global maximum) such as Simulated Annealing (SA) [13], if no other constraint on the solution are set. Anyway, SA can be excessively time demanding. In order to produce *quasi* real-time field maps to correct images during acquisition, long computation time is a big disadvantage of SA algorithm which limits its applicability.

To overcome this problem, the optimization algorithm that we use in this paper is based on the Graph Cut theory [14,15]. The main feature of graph cut optimization algorithms is that they are able to provide the global maximum or a local one within a good quality, without being computational time demanding.

Graph Cut theory has already been applied in the MRI field by Hernando *et al.* [16]. Differently from [16], we use the graph cut optimization algorithm proposed by Ishikawa [14] which, if the graph is correctly constructed and if some hypothesis are respected while constructing it, is able to provide the global maximum of the considered function. The hypotheses at the basis of the Ishikawa algorithm are two: the first one is related to the convexity of the *a priori* energy. This hypothesis is respected in our model, since we are using the TV model. The second hypothesis is related to the linear order of the *label set*. The label set is the set of all the possible values that the pixels of the image can assume. For the problem considered in this paper, the labels correspond to the Lamor frequencies ***ω***. To satisfy this condition we suppose that Lamor frequencies *ω* can be represented as integers in the range between 1 and *K*, where *K* is the size of the label set.

The Ishikawa algorithm is based on computing a minimum cut in a particular graph. The graph *G* = (*V*, *E*) contains *V* = *P* × *K* vertexes + 2 special vertexes *S* and *T* (the *source* and the *sink*). We recall that *P* is the number of the pixels of the image. A vertex is the intersection of a pixel value and of a label value of the graph and is indicated with the following notation: *v*(*p*, *k*) where *p* is referred to the *p*-th pixel and *k* to the *k*-th label.

**Figure 2.**
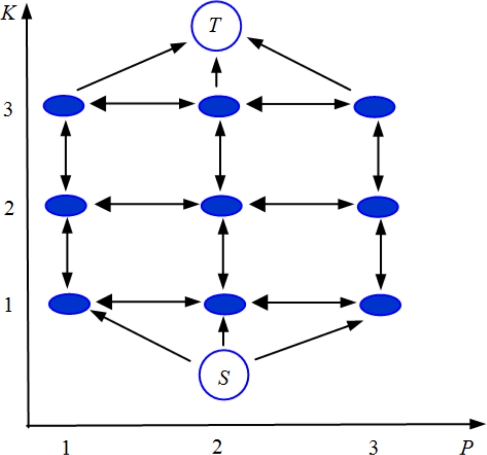
One dimensional Ishikawa graph construction: circles represent the vertexes *V*, arrows represent the edges *E*.

A simplified representation of the Ishikawa graph for the 1 dimensional case is shown in Figure 2. The *V* vertexes (the circles in Figure 2) are connected by the edges *E* (the arrows on Figure 2). Three families of edges are created: *data edges* (vertical arrows going up), *constraint edges* (vertical arrows going down) and *interaction edges* (horizontal arrows).

Each of the edges has a certain *capacity c.* In the Ishikawa graph the vertical edges are related to the likelihood function while the horizontal ones take into account the *a priori* information. In particular the capacity of the data edge between the vertex *v*(*p*,*k*) and the vertex *v*(*p*,*k*+1) is set using the following equation:

(14) $$\{\begin{array}{l} c(v(p,k),v(p,k+1))=L\left({\overset{⌣}{\phi }}_{p}|\omega_{p}(k)\right)\\ c(S,v(p,1))=\infty \end{array}$$

which represents the multi-acquisition likelihood value, calculated from Equation (10) considering the *p*-th pixel and frequency value *ω_p_* = *k*. The constraint edges are set to be infinity in order to cut only one label for each pixel. The interaction edges are related to the *a priori* energy function. Using the TV model it can be shown that the capacity of the horizontal edge between the vertex *v*(*p*,*k*) and the vertex *v*(*p +* 1,*k*) is set using the following Equation [14]:

(15) c(v(p,k),v(p+1,k))=1

Note that the proposed *a priori* TV model allows us to reduce the computational cost of the Ishikawa optimization method since it reduces the number of interaction edges of the graph [14].

A minimum cut on the graph consists of separating the special vertexes *S* and *T* by minimizing the sum of the capacities relative to cut edges. This is equivalent to find the solution of our maximization problem (*i.e.*, the solution of Equation (13)). Thus, finding a minimum cut on the Ishikawa graph, given the capacities of Equations (14) and (15), corresponds to reconstruct the Larmor angular frequencies of the whole images and consequently the field map. The minimum cut is computed using the Max Flow algorithm (the code by V. Kolmogorov is available at http://www.cs.ucl.ac.uk/staff/V.Kolmogorov/software.html).

## 5. Results and Discussion

In this section, some case studies are presented in order to show the performances achievable by the presented method. Results are obtained applying the method to both simulated and real data sets. For all the presented cases, a constant magnetic field of *B*_0_ = 1.5 T, corresponding to a central Larmor angular frequency of *ω* = 4.0127 × 10^8^Hz, and different echo time and SNR configurations are considered. The size of the images is set to be 128x128 pixels and we use *K* = 150 labels. In all the presented cases, to perform automatic regularization parameter estimation *β*, we used the method based on the *L*-curve, in particular the triangular method described in [17].

**Figure 3.**
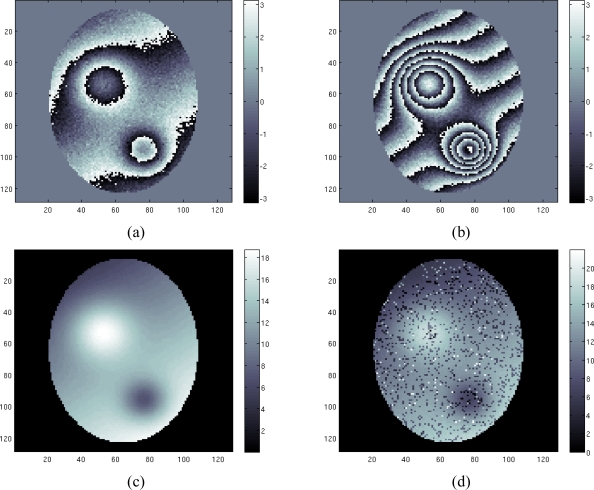
First case study: (a) first phase image, (b) fourth phase image, (c) estimated field map using proposed technique, (d) estimated field map using conventional ML technique.

In the first case study a discontinuity free field map is estimated considering four images with SNR = [6 5 4 3] dB and echo times *T_E_* = [3.3 5.7 8.1 10.5] msec. Figures 3(a) and (b) show the phase of the first image (acquired at the lowest *T_E_*) and fourth image (acquired at the highest *T_E_*).

As expected, the phase of the first image presents fewer fringes than the other one. We apply to the four acquisition data set our proposed approach based on the maximization of Equation (13) using the Ishikawa algorithm. The results are shown in Figure 3(c). As we can see the reconstruction is performed well. The unwrapping problem has been successfully solved, and the field map is retrieved. Note that the algorithm is in the same time able to solve the unwrapping problem and to restore the solution (*i.e.*, to remove the noise from the reconstructed field map). Figure 3(d) shows the reconstruction of the field map using a conventional Maximum Likelihood (ML) estimation. Note that the noisy data have been masked before the estimation, by thresholding the coherence map.

In order to evaluate the advantage of the multi-acquisition configuration, we perform the reconstruction using only two and three acquisitions, instead of four. The results of the reconstruction in terms of normalized mean square errors are shown in Table 1. As we can see, using more acquisitions allows the method to improve its performances, thus providing a better reconstruction.

**Figure 4.**
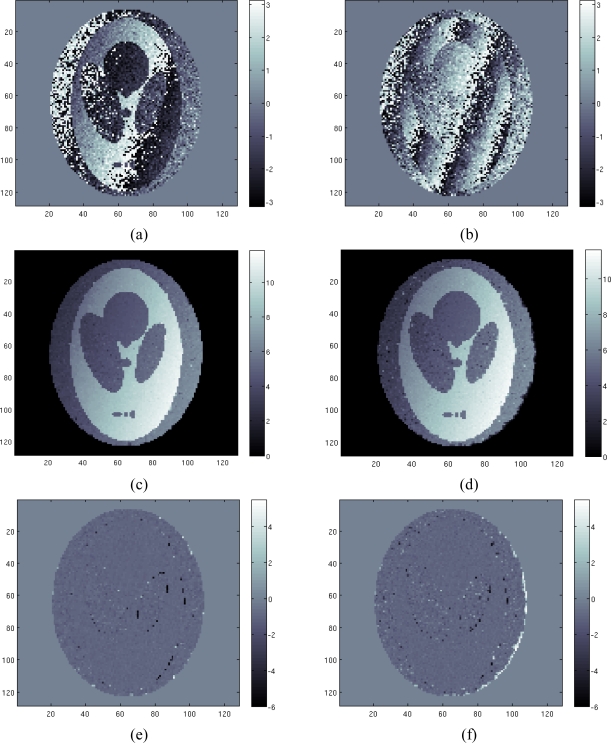
Second case study: (a) first phase image, (b) fourth phase image, (c) estimated field map using proposed method, (d) estimated field map using the approach of paper [4], (e) difference between estimated and the true field map using proposed method, (f) difference between estimated and the true field map using the approach of paper [4].

For the second case study a simulated scenario with air/tissue discontinuities is considered. This simulation, although not completely realistic in electromagnetic terms of the magnetic field local strength, is shown to remark the robustness of the proposed algorithm in tackling the phase unwrapping problem when discontinuities are present. Configuration parameters are the same used in the previous case, in a higher noise case. As before, we use four different acquisitions (four phase images). In Figures 4a,b we show the least wrapped and the most wrapped phase images. Note that in this case the simulation takes into account the effects at the air/tissue interfaces, where strong changes of the field map are localized. Differently from the previous case, this time the field map can be very difficult to be retrieved using classical approaches, due to the presence of the discontinuities that make the unwrapping problem a hard task. We apply to the four acquisition data sets the proposed algorithm. The reconstructed profile is shown in Figure 4(c). Once again the reconstruction is very satisfactory. The field map is well reconstructed in all tissue areas. We can note the good behavior of the TV model, which is able in to preserve edges, without penalizing smooth areas. Figure 4(d) shows the estimated field map using the approach proposed in [4]. Comparing Figures 4e,f, representing the reconstruction error map of our approach and the approach of [4] respectively, it can be noted the better accuracy of the first one compared to the latter. In particular, we can see that our approach provides a less noisy reconstruction compared to the approach presented in [4] and it better handles discontinuities at air/tissue interfaces. This is confirmed from the computation of the normalized mean square error, which is equal to 0.039 and to 0.043 for our approach and for the approach of [4] respectively. Note that for both reconstructions, the best trade off between under regularization and correct discontinuities retrieving has been used. Anyway we can remark also a known drawback of the TV model, which consists of the loss of the contrast in the reconstructed image. The reconstructed field map is well reconstructed but it is a little bit over regularized.

Also in this case, we perform the reconstruction using only two and three acquisitions, instead of four. The results of the reconstruction in terms of normalized mean square errors are shown in Table 2. The trend seen for the previous example is respected. Using more acquisitions allows the method to improve its performances, thus providing a better reconstruction.

It is interesting to compare Table 1 and Table 2, to appreciate the effectiveness of the multi-acquisition approach when dealing with discontinuities. As a matter of fact, increasing the number of used acquisitions in the second study case (Table 2) improves, proportionally, more the reconstruction performances respect to the first study case (Table 1).

As a last study case, we consider the same study case of the second one, but with more noisy data (SNR lowered of 2.5 dB). This time the SNR is set to be [3.5 2.5 1.5 0.5] dB. The results of the estimation are show in Table 3 in terms of normalized mean square error.

As expected, the error increases compared to the second study case, but, even in presence of noisy data, the algorithm is able to provide a good solution. We underline that for all the three case studies the proposed algorithm is able to provide the solution in less than one minute using a SUN Ultra 40 Workstation.

**Figure 5.**
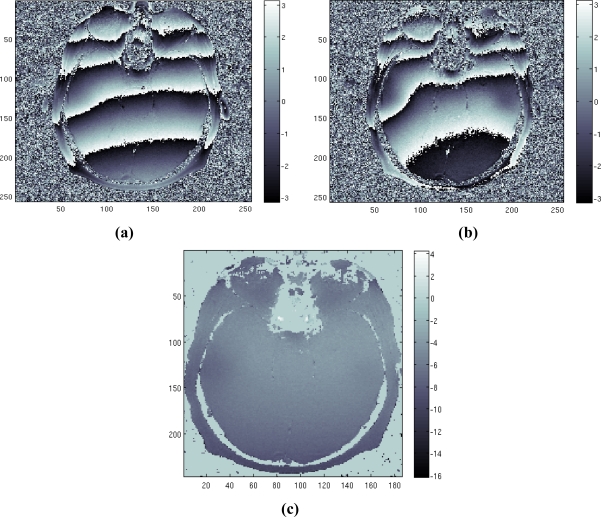
Real case study: (a) first phase image, (b) second phase image, (c) estimated field map.

Finally, we test the method on a real data set. The data set consists of two head images acquired in axial position with echo times equal to *T_E_* = [12.8 25.6] msec. Note that the ratio between the two *T_E_* values is a natural value, which is the worst case for our method. The images were taken from the Radiological Sciences Laboratory, Stanford University, School of Medicine. Figures 5a,b shows the phase of the two available images. Figure 5(c) represents the estimated field map obtained applying our method, proving the effectiveness of the method.

Note that in all reconstructions only signal relative to water component of the tissue is considered. The presented method can be applied also in case of superposed fat component signal. Under the hypothesis of selecting echo times in order to obtain in phase superposition of the two components, fat signal becomes undetectable and does not influence the field map estimation. In our approach this is possible since we are not limited in the range and the spacing of *T*_E_ we have to use.

## 6. Conclusions

In this paper a novel approach for the field map estimation problem in Magnetic Resonance Imaging has been presented. The main characteristics of proposed method are the statistical approach and the fast optimization algorithm based on Graph Cuts. The algorithm has shown to correctly retrieve the field map in a wide range of scenarios, both on simulated and real data. It is able to solve the phase unwrapping problem and to work properly both with low and high SNRs as with different echo times. We have shown that the approach is able to correctly manage the sharp discontinuities that arise at air/tissue boundary. Moreover, due to the piecewise smooth nature of field maps, the proposed *a priori* model, the TV model, has shown to be effective since it allows us to correctly reconstruct both smooth areas and field discontinuities. Two final interesting remarks about the use of Graph Cuts optimization procedure: first, it is characterized by low computational time, allowing quasi real-time field map estimations; secondly it ensures to reach the global optimum solution. A next step will be the evaluation of the method’s performance in real clinical MRI applications.

## Figures and Tables

**Table 1.** Normalized mean square error for different number of available acquisitions—discontinuities free field map case.

2 acquisitions (***T****_E_* = [3.3 5.7])	0.0117
3 acquisitions (***T****_E_* = [3.3 5.7 8.1])	0.0098
4 acquisitions (***T****_E_* = [3.3 5.7 8.1 10.5])	0.0091

**Table 2.** Normalized mean square error for different number of available acquisitions—air/tissue discontinuities field map case.

2 acquisitions (***T****_E_* = [3.3 5.7])	0.1010
3 acquisitions (***T****_E_* = [3.3 5.7 8.1])	0.0685
4 acquisitions (***T****_E_* = [3.3 5.7 8.1 10.5])	0.0392

**Table 3.** Normalized mean square error for different number of available acquisitions—low SNR case.

2 acquisitions (***T****_E_* = [3.3 5.7])	0.1634
3 acquisitions (***T****_E_* = [3.3 5.7 8.1])	0.1028
4 acquisitions (***T****_E_* = [3.3 5.7 8.1 10.5])	0.0880
